# Intentional intoxication with monkshood plant leading to atrioventricular dissociation and ventricular ectopy in a 17-year-old female: a case report

**DOI:** 10.1186/s12245-024-00588-3

**Published:** 2024-02-08

**Authors:** V. W. Klokman, S. Tempelaar, B. C. W. Kuipers, I. A. G. van Dijk, M. A. M. Moviat

**Affiliations:** 1 Department of Vascular Surgery, University Medical Center, Utrecht, The Netherlands; 2 Department of Emergency Medicine, Jeroen Bosch Hospital, ‘S-Hertogenbosch, The Netherlands; 3 Department of Pediatrics, Jeroen Bosch Hospital, ‘S-Hertogenbosch, The Netherlands; 4 Department of Intensive Care Medicine, Jeroen Bosch Hospital, ‘S-Hertogenbosch, The Netherlands

**Keywords:** Monkshood plant, Aconitine, Poisoning, Arrythmia, AV dissociation, Emergency medicine

## Abstract

**Background:**

Monkshood, a toxic plant containing a potent cardio- and neurotoxin called aconitine, can lead to a range of symptoms, including nausea, vomiting, dizziness, seizures, and cardiac arrhythmias. Mortality associated with this intoxication are due to ventricular tachyarrhythmias which are difficult to treat and often refractory in nature.

**Case presentation:**

We present a case of a 17-year-old female patient who presented to the emergency department after intentionally ingesting a monkshood plant and developed atrioventricular dissociation and frequent ventricular ectopy. The patient was successfully treated with activated charcoal, supportive care, and cardiac monitoring.

**Conclusion:**

This case highlights the importance of early recognition of aconitine poisoning and the need for prompt supportive care, cardiac rhythm monitoring, and preemptive antiarrhythmic treatment planning.

## Background

*Aconitum* sp., also known as monkshood or wolfsbane, is a plant containing aconite, a highly toxic alkaloid found in the roots, stem, leaves, and seeds. Aconite acts by blocking the sodium channels in excitable tissues in the myocardium, nerves, and muscles resulting in tissue depolarization [1, 2]. Exposure to aconite may cause gastrointestinal symptoms, such as nausea, vomiting, and abdominal pain, followed by neurological symptoms such as paresthesia’s, and ultimately potentially lethal cardiac sequela [1]. A broad spectrum of arrhythmias are possible, though the clinical presentation of aconitine poisoning can vary depending on the dose ingested and the route of administration. We present a case of a 17-year-old female patient who presented to the emergency department (ED) after intentionally ingesting the monkshood plant and developed an accelerated junctional rhythm with atrioventricular dissociation with ventricular ectopy.

## Case presentation

A 17-year-old female with a past medical history of depression and two previous autointoxications. Current medication use included once daily use of fluoxetine at a dosage of 5 mg. The patient presented to the emergency department (ED) at 6:37 pm with complaints of nausea, vomiting, and dizziness after intentionally ingesting an unknown amount of monkshood plant at approximately 5:00 pm. After a fight with her parents, the patient became depressed with suicidal ideologies. Having recently watched the television series “Ginny and Georgia,” she saw that a character poisoned another character using a certain plant. The patient researched the episode online and was able to identify and purchase the plant (*Aconitum napellus*) at her local garden center. At home, she pureed the plant’s leaves, flowers, and stem and drank it in a smoothie. Several minutes afterwards, she began to experience severe tingling sensations and paresthesia in her entire body. Subsequently, she developed stomach pain, causing her to rethink her earlier suicidal path, and thus, she presented to the ED on her own volition 1.5 h after ingestion.

**Fig. 1:**
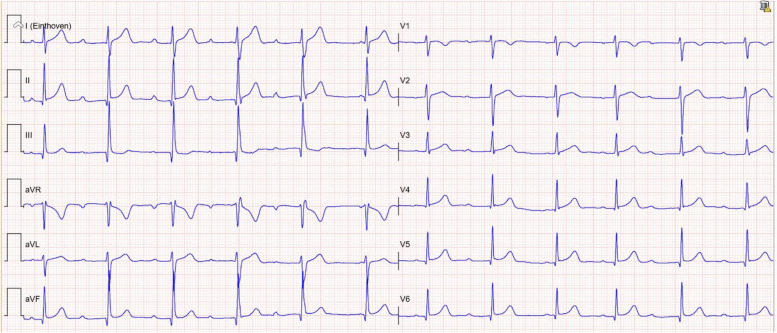
Initial electrocardiogram upon emergency department arrival (1.5 h after ingestion) showing varying PR times with signs of AV dissociation and an accelerated junctional rhythm

**Fig. 2:**
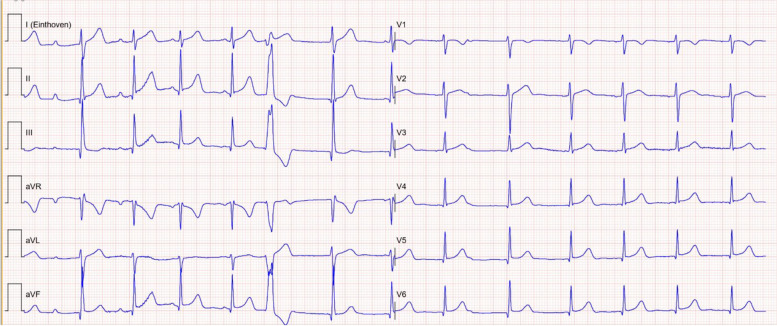
Second electrocardiogram made in the emergency department (2 h after ingestion) showing varying PR times with signs of AV dissociation, an accelerated junctional rhythm, and ventricular ectopy

**Fig. 3:**
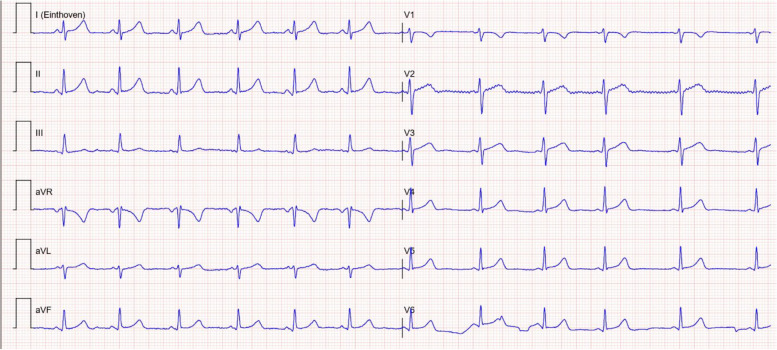
Electrocardiogram 12 h after admission (13.5 h after ingestion) with now normalization to a sinus rhythm

At the ED, physical examination and laboratory analysis (Table 1) were normal. Serial electrocardiograms (ECG) were made where an accelerated junctional rhythm with atrioventricular dissociation (Fig. 1) and ventricular ectopy (Fig. 2) was seen. The plant was confirmed by garden center purchase receipt with corroboration with the National Poison Control Center. Immediately upon arrival to the ED, the patient was rapidly treated with activated charcoal with laxatives, which she received multiple times over the course of her admission. The patient was admitted to the intensive care unit (ICU) for observation and monitoring of her cardiac rhythm. The following day after initial admission, her cardiac rhythm normalized (Fig. 3) at approximately 12 h post ingestion with only supportive care.

**Table 1:** Laboratory findings on emergency department arrival

	**Value (normal range)**	**Units**
**Complete blood count**		
Hemoglobin	8.3 (7.5–10.0)	mmol/L
Red blood cells	4.47 (4.00–5.00 × 10^12^)	/L
White blood cells	5.7 (4.0–10.0 × 10^9^)	/L
Platelets	284 (150–400 × 10^9^)	/L
**Biochemistry**		
Sodium	141 (135–145)	mmol/L
Potassium	4.2 (3.5–4.8)	mmol/L
Chloride	105 (97–107)	mmol/L
Calcium	2.41 (2.15–2.60)	mmol/L
Magnesium	0.86 (0.70–1.10)	mmol/L
Blood urea nitrogen	3.5 (2.5–6.4)	mmol/L
Creatinine	63 (45–71)	µmol/L
Total protein	71 (63–83)	g/L
Albumin	44 (35–50)	g/L
Creatinine kinase	191 (0–144)	U/L
Aspartate transaminase	22 (0–29)	U/L
Alanine transaminase	41 (0–34)	U/L
Lactate dehydrogenase	217 (0–249)	U/L
Alkaline phosphatase	89 (40–160)	U/L
Gamma-glutamyltransferase	17 (0–39)	U/L
Total bilirubin	14 (0–16)	µmol/L
**Venous blood gas**		
pH	7.38 (7.32–7.43)	
PaCO_2_	5.9 (5.4–7.0)	kPa
PaO_2_	3.5	kPa
HCO_3−_	26 (23–30)	mmol/L
Base excess	0.4	mmol/L

## Discussion

*Aconitum* sp., commonly known as monkshood, wolfsbane, or aconite, is a commonly found plant in Europe, North America, and Asia [2]. Its root is commonly used in traditional Chinese medicine and ayurvedic medicine for analgesic, anti-inflammatory, and cardiotonic regimens [1]. A degree of processing is necessary to reduce alkaloid content, though many reports of poisoning have been reported to due incorrect preparation and application [3, 4]. Mass poisoning has also been reported in the case of food contamination [4]. Other at-risk groups for poisoning include nature-going patients as they may mistake the plant for an edible variety [5].

*Aconitum* species contain high concentrations of alkaloids (aconitine, mesaconitine and hypaconitine) that have toxic effects on neurons, the myocardium, and muscle cells [2]. The toxicity occurs through the high-affinity binding capabilities of aconitine to voltage-gated sodium channels (VGSC), causing cell depolarization and permanent activation of these channels [1]. This subsequently produces higher sodium concentrations in the cardiac cells, which may induce life-threatening arrhythmia’s [3]. A broad range of tachyarrhythmia’s are possible from ventricular tachycardia, torsade de pointes, and ventricular fibrillation to bradyarrhythmia’s such as junctional rhythm, fascicular and bundle branch blocks, or repolarization abnormalities with QT prolongation [1, 6]. Neurological complications include diaphoresis, obnubilation, blurred vision, color distortion, weakness, tingling, incoordination of extremities, and muscular paralysis that can result in respiratory arrest, all caused by permanent activation of VGSC [5]. The clinical presentation of aconitine poisoning can vary dependent on the dose ingested and the route of administration. Clinical symptoms may persist for up to 30 h, while a half-life of aconitine has been reported to be about 3 h [7, 8].

There is no specific antidote for aconitum poisoning and thus no specific treatment strategy. The treatment mainstays are similar to all vegetal intoxications and involve decreasing the absorption utilizing activated charcoal with laxatives, supportive care, and symptom management [9]. Activated charcoal may be given multiple times and up to 6 h after ingestion to reduce enteral aconitine uptake [9]. The greatest risk to the patient is ventricular tachyarrhythmia which are often refractory to both cardioversions and antiarrhythmic drugs [1]. Therefore, a predetermined strategy for the treatment of these potential and complex rhythm disturbances is of importance.

There is no consensus of the best antiarrhythmic treatment strategy for intoxication related ventricular tachyarrhythmia’s in both adults and children. A case series by Coulson et al. (2017) compiled the outcomes of 65 case reports and the treatment strategies employed [10]. The most used and effective therapies were flecainide and amiodarone when compared to lidocaine and cardioversion in returning the patient to normal sinus rhythm, though sporadic use of mexiletine, procainamide, and magnesium sulfate also saw a small degree of effectiveness [10]. In the pediatric population, amiodarone use is debated as there is relatively few data on the safety and efficacy in children with the potential for adverse effects to occur [11]. Cardiopulmonary resuscitation and cardiopulmonary bypass may be of use in order to buy time for the body to naturally excrete the alkaloids if the patient is non-responsive to either chemical or electric cardioversion, a strategy that was successful in 4 out of 6 patients [10]. However, despite these efforts, a mortality rate of 15% was seen in patients that develop refractory tachyarrhythmia’s [10].

## Conclusion

This case highlights the importance of early recognition of aconitine poisoning and the need for prompt supportive care and monitoring of cardiac rhythm. Ingestion of monkshood plant can lead to a range of symptoms, including life-threatening refractory cardiac arrhythmias. Admitting ED physicians should be aware of the potential sequelae of aconitine poisoning and account for preemptive antiarrhythmic treatment strategies. Literature review suggests that either flecainide or amiodarone presents the high chance for successful return to normal sinus rhythm in the event of tachyarrhythmia’s. Patient disposition should be to medical units (i.e., ICU or cardiac units) that provide intensive cardiac monitoring to allow for the best outcomes.

## Data Availability

All data generated or analyzed during this study are included in this published article (and its supplementary information files).
